# Editorial: Neuroimaging in psychiatry 2023: schizophrenia

**DOI:** 10.3389/fpsyt.2025.1591274

**Published:** 2025-04-21

**Authors:** Massimo Tusconi, Serdar M. Dursun

**Affiliations:** ^1^ Department of Medical Sciences and Public Health, University of Cagliari, Cagliari, Italy; ^2^ Neurochemical Research Unit, Department of Psychiatry, University of Alberta, Edmonton, AB, Canada

**Keywords:** neuroimaging, biomarkers, neurobiology, schizophrenia, psychosis, machine learning

Schizophrenia is a complex mental condition that affects approximately 1% of the World’s population (1). Despite extensive research, the neurobiological mechanisms underlying schizophrenia remain poorly understood (1, 2). It is clear that schizophrenia remains one of the most complex and distressing psychiatric disorders, with a clinical picture characterized by a series of positive symptoms (hallucinations, delusions) (3), negative symptoms (diminished affect, social withdrawal) and cognitive impairments (4–6). Alongside the most significant advances in neurobiological research, it is evident that voxel-based morphometry and high-resolution diffusion imaging have revealed widespread reductions in gray matter volume (GMV) and white matter integrity (7–9). It is recognized that structural deficits, predominantly in the prefrontal cortex, temporal lobes and striatum, are often correlated with disease severity and cognitive decline; in particular, early-onset schizophrenia presents more pronounced structural abnormalities, offering unique insights into its pathophysiological mechanisms (10). Functional imaging studies show reduced activation in the anterior insula and anterior cingulate cortex during socio-cognitive tasks, suggesting a disturbed integration of emotional and cognitive processes; these results are in line with behavioral observations of impaired social functioning, emphasizing the need for targeted and tailored therapeutic interventions with a view to precision medicine (11–13). Machine learning approaches have perfected the prediction of treatment outcomes in schizophrenia (3). Multimodal imaging biomarkers, incorporating structural and functional data, demonstrate high accuracy in identifying treatment-resistant schizophrenia (TRS) and in predicting disease trajectories (14, 15). Individualized network parcellation and advanced classification algorithms achieve robust performance, with predictive accuracies exceeding 90% in some studies; moving forward in this direction, an increasingly exploratory literature on the integration of neuroimaging, computational models, and large-scale data repositories suggests a new era in schizophrenia research (16, 17). Transdiagnostic frameworks that transcend traditional diagnostic boundaries are set to improve our understanding of common and distinct pathophysiological features, facilitating the differential diagnosis of bipolar disorder, and ultimately reducing the stigma associated with the disease and paving the way for integrated management of these clinical aspects (18–20), with a view to increasingly multidisciplinary rehabilitation through the use of virtual reality techniques, stimulation of mirror neurons, achieving better outcomes with a reduction in the invasiveness of treatments (21–23). Recent advances in neuroimaging techniques may provide new insights and allow for highly specific information on the neural correlates of schizophrenia (5, 24). In this perspective, this Research Topic aims to highlight the latest discoveries in the field of neuroimaging and explore their potential implications for the diagnosis and treatment of schizophrenia, while providing a valuable resource for healthcare professionals, from both a clinical and an etiopathogenetic point of view (25).

Regarding the analysis of the neurobiological heterogeneity of the clinical state at high risk for psychosis, Oliver et al. present a study based on a large neuroimaging dataset of individuals at clinical high risk for psychosis (CHR-P) who meet the brief limited intermittent psychotic symptoms (BLIPS) criteria obtained by combining data from four independently conducted studies. The authors found weak or moderate evidence of no differences in global gray matter (GM), regional cerebral blood flow (rCBF), hippocampal and striatal attenuation of psychotic symptoms (APS) and BLIPS, suggesting based on their results that rCBF alone may not be suitable for risk stratification in CHR-P subjects.

In their study on the differentiation of the trajectories of retinal morphological aging in schizophrenia and their associations with cognitive dysfunction, Domagała et al. demonstrate that, in patients suffering from schizophrenia, the retinal macula undergoes accelerated atrophy starting from the third decade of life, similar to the dynamics of white matter changes analyzed in relation to the hypothesis of accelerated aging. The curves indicating age-related changes in other retinal structures were generally very similar in both groups, only with more pronounced thinning in the patient samples, with associations between the macula, ganglion cell complex and the age of the patients affecting only the middle-aged subgroup, suggesting on the basis of the data presented that retinal abnormalities in schizophrenia do not increase linearly over the course of life.

Additionally, in an analysis of white matter tracts in schizophrenia, bipolar disorder, aging and dementia using high spatial and directional resolution image diffusion, Mamah et al. provide preliminary data comparing image diffusion metrics between younger psychiatric populations and older cohorts using an automated trait-based analysis. The study shows that white matter tract volumes did not differ significantly between the groups evaluated, while there were significant differences in fractional anisotropy of the tracts in the various tracts studied. The authors, using an automated tractography tool, showed white matter integrity significantly compromised with aging, suggesting demyelination.

Shifting the focus to empathy in schizophrenia, Knobloch et al. analyze the neural alterations during emotion recognition and affective sharing. From a behavioral point of view, the patients only showed a prolonged response time in the age discrimination tests, while in the emotion processing tests, the patients showed a difference in neural response, without an observable behavioral correlate. The study suggests that the patients have deficits in processing complex visual information regardless of the emotional content at a behavioral level, and that these deficits coincide with aberrant neural activation patterns in the emotion processing networks.

Furthermore, in their systematic review Merola et al. examine transdiagnostic markers in the psychosis continuum, highlighting results that provide preliminary evidence for potential transdiagnostic alterations in brain activity in specific regions associated with psychosis, although they are not confirmed by survival to correction for multiple comparisons.

In an in-depth study of illness-related variables and resting-state brain activity abnormalities in schizophrenia, Giuliani et al. emphasize how attention/vigilance deficits were negatively associated with the dorsal resting-state (RS) activity of the anterior cingulate and, together with depression, were positively associated with the RS activity of the right dorsolateral prefrontal cortex. These deficits, along with the impairment of reasoning/problem-solving and conceptual disorganization, were associated with RS activity of the right inferior parietal lobe and right parietal temporal junction, highlighting how neurocognitive deficits and negative symptoms are associated with different neural markers.

Delving into brain structure, Zhang et al. present a study describing how individualized multimodal biomarkers obtained from magnetic resonance imaging predict the one-year clinical outcome in first-episode, non-medicated patients with schizophrenia; the study evaluated the structural morphology and functional topological characteristics related to treatment response using an individualized parcellation analysis in combination with machine learning (ML). This allowed us to highlight the potential of individual-specific network parcellation in the prediction of treatment-resistant schizophrenia, emphasizing the crucial role of feature attributes in the accuracy of the predictive model.

Finally, Wang et al. provide a meta-analysis of structural and functional brain abnormalities in early-onset schizophrenia; their work revealed that certain regions in the EOS showed significant structural or functional abnormalities, such as the temporal gyri, prefrontal cortex and striatum. These results may help to deepen our understanding of the pathophysiological (26) mechanisms underlying EOS and provide potential biomarkers for the diagnosis or treatment of EOS.

In conclusion, although progress in neuroimaging is extremely promising, challenges remain, including the need for larger and more diverse data sets and ethical considerations regarding data privacy (27). Despite these limitations, the integration of neuroimaging with computational methods continues to shape the future of schizophrenia research, fostering a deeper understanding of this enigmatic disorder (28). A combined approach based on neuroimaging, the latest machine learning techniques and evidence of improvements in the final outcome of the most modern rehabilitative integrations with the most traditional methods, is leading us toward emerging transdiagnostic frameworks that challenge traditional diagnostic boundaries and support a continuum-based approach to psychosis (29). Neuroimaging studies reveal pathophysiological characteristics common to all conditions, such as interrupted connectivity and abnormalities in neurological development (30). These insights pave the way for integrative models that take into account genetic, environmental and developmental factors, filling in the gaps in our understanding and ultimately enabling us to achieve results that pave the way for future innovations in the research and treatment of psychosis.
